# DJ-1 Inhibits α-Synuclein Aggregation by Regulating Chaperone-Mediated Autophagy

**DOI:** 10.3389/fnagi.2017.00308

**Published:** 2017-09-27

**Authors:** Chuan-Ying Xu, Wen-Yan Kang, Yi-Meng Chen, Tian-Fang Jiang, Jia Zhang, Li-Na Zhang, Jian-Qing Ding, Jun Liu, Sheng-Di Chen

**Affiliations:** ^1^Department of Neurology and Collaborative Innovation Center for Brain Science, Ruijin Hospital, School of Medicine, Shanghai Jiao Tong University, Shanghai, China; ^2^Laboratory of Neurodegenerative Diseases, Institute of Health Sciences, Shanghai Institutes for Biological Sciences, Chinese Academy of Sciences, University of Chinese Academy of Sciences, Shanghai, China; ^3^Department of Biostatistics, School of Medicine, Shanghai Jiao Tong University, Shanghai, China

**Keywords:** Parkinson’s disease, α-synuclein, DJ-1, chaperone-mediated autophagy, LAMP2A

## Abstract

α-Synuclein misfolding and aggregation play an important role in the pathogenesis of Parkinson’s disease (PD). Loss of function and mutation of the PARK7/DJ-1 gene cause early-onset familial PD. DJ-1 can inhibit α-synuclein aggregation, and may function at an early step in the aggregation process. Soluble wild-type (WT) α-synuclein is mainly degraded by chaperone-mediated autophagy (CMA), and impairment of CMA is closely related to the pathogenesis of PD. Here, we investigated whether DJ-1 could reduce α-synuclein accumulation and aggregation by CMA. DJ-1 knockout mice and DJ-1 siRNA knockdown SH-SY5Y cells were used to investigate the potential mechanisms underlying the relationship between DJ-1 deficiency and α-synuclein aggregation. First, we confirmed that DJ-1 deficiency increased the accumulation and aggregation of α-synuclein in both SH-SY5Y cells and PD animal models, and overexpression of DJ-1 *in vitro* effectively decreased α-synuclein levels. α-Synuclein overexpression activated CMA by elevating the levels of lysosome-associated membrane protein type-2A (LAMP2A), but DJ-1 deficiency suppressed upregulation of LAMP2A. DJ-1 deficiency downregulated the level of lysosomal 70 kDa heat-shock cognate protein (HSC70) but not the levels of that in homogenates. Further studies showed that DJ-1 deficiency accelerated the degradation of LAMP2A in lysosomes, leading to the aggregation of α-synuclein. Our study suggests that DJ-1 deficiency aggravates α-synuclein aggregation by inhibiting the activation of CMA and provides further evidence of the molecular interaction between PD-related proteins via the CMA pathway.

## Introduction

Loss-of-function mutations of the PARK7/DJ-1 gene cause early-onset familial Parkinson’s disease (PD). PD is one of the most common progressive neurodegenerative disorders and is characterized by the loss of dopaminergic neurons in the substantia nigra pars compacta and the presence of α-synuclein-containing Lewy bodies in surviving neurons (Spillantini et al., 1997, 1998; Lees et al., 2009). Recently, DJ-1 has received extensive attention for its intimate relationship with both familial and sporadic PD (Choi et al., 2006).

DJ-1 is a highly conserved and extensively expressed protein that is involved in various cellular processes, such as anti-oxidative stress, chaperone functions, transcriptional regulation, protease activities and mitochondrial regulation (Ariga et al., 2013; Trempe and Fon, 2013). DJ-1 inhibits α-synuclein aggregate formation but does not colocalize with α-synuclein aggregates (Shendelman et al., 2004), suggesting that DJ-1 may play an inhibitory role in the early stage of α-synuclein aggregates consisting of misfolded monomers but not oligomers, fibrils, or even Lewy bodies.

Point mutations (A53T, A30P and E46K; Polymeropoulos et al., 1997; Krüger et al., 1998; Zarranz et al., 2004) or multiplications (Chartier-Harlin et al., 2004) of the α-synuclein gene locus result in autosomal-dominant familial PD, whereas polymorphisms within the α-synuclein locus (Simón-Sánchez et al., 2009) confer an increased risk of sporadic PD. These findings demonstrate the critical role of increased levels of α-synuclein with the exception of gene mutations in the pathogenesis of PD. α-Synuclein levels in the central nervous system rely on the balance between α-synuclein synthesis and degradation (Vekrellis et al., 2011). Lysosomal impairment supposedly plays a critical role in the aggregation of α-synuclein and the pathogenesis of PD (Moors et al., 2016). Depending on the mechanisms of cytoplasmic substrates delivery to the lysosome, the autophagy-lysosome pathway can be divided into three types, macroautophagy, microautophagy and chaperone-mediated autophagy (CMA; Cuervo and Wong, 2014). Macroautophagy is mainly responsible for the removal of oligomeric α-synuclein (Lee et al., 2004), whereas CMA predominantly degrades wild-type (WT) monomeric α-synuclein (Cuervo et al., 2004; Vogiatzi et al., 2008).

CMA, a selective form of the autophagic pathway, is responsible for the lysosomal degradation of cytosolic proteins with a targeting motif (Kaushik and Cuervo, 2009), which can be recognized by the 70 kDa heat-shock cognate protein (HSC70) co-chaperone complex (Chiang et al., 1989; Dice, 1990). Once recognized, cytosolic HSC70 (cyt-HSC70) delivers the substrate to the lysosomal surface and binds to the lysosome-associated membrane protein type 2A (LAMP2A; Cuervo and Dice, 1996). After binding, LAMP2A can multimerize to form the translocation complex (Bandyopadhyay et al., 2008), and substrates can unfold and translocate across the LAMP2A translocation complex before arriving at lysosomal lumen, where they are quickly degraded by the proteases (Bejarano and Cuervo, 2010). WT α-synuclein is a substrate of CMA (Cuervo et al., 2004; Vogiatzi et al., 2008), and CMA dysfunction may generate an increase in pathological α-synuclein aggregates, which in turn may further block both their own degradation and that of other CMA substrates, thus leading to a vicious cycle of neurotoxicity (Cuervo et al., 2004). This dual role of CMA in neurodegenerative disorders led us to investigate whether DJ-1 could inhibit α-synuclein accumulation and aggregation by regulating CMA.

Here, DJ-1 deficiency intensified α-synuclein accumulation and aggregation in SH-SY5Y cells and PD animal models. α-Synuclein overexpression activated CMA by elevating the levels of LAMP2A, but DJ-1 deficiency suppressed upregulation of LAMP2A. Further studies showed that DJ-1 deficiency accelerated the degradation of LAMP2A in lysosomes, leading to the aggregation of α-synuclein.

## Materials and Methods

### Reagents

Methyl-4-phenyl-1,2,3,6-tetrahydropyridine (MPTP, M0896), ammonium chloride (NH_4_Cl, A9434), Chloroquine diphosphate salt (CQ, C6628), 3-methyladenine (3-MA, M9281) and cycloheximide (CHX, C7698), proteinase K (P2308) were purchased from Sigma. Lactacystin (BML-P1104-0200) was purchased from Enzo. Allstars Negative Control siRNA (scramble, 1027280), siRNA for LAMP2 (1027416/GS3920) and siRNA for DJ-1 (1027416/GS11315) were purchased from Qiagen. SiRNA for LAMP1 (A10001) and scramble were purchased from GenePharma. Cell Premix Ex Taq (Til RNaseH Plus) was purchased from Takara (RR420B). A Lysosome Enrichment Kit for Tissue and Cultured Cells (89839) and Protein Extraction Reagent Kit (23225) were purchased from Thermo Scientific. Phenylmethylsulfonyl fluoride (PMSF, ST 506) and 3-(N-Morpholino) propanesulfonic acid (MOPS, ST302) was purchased from Beyotime Biotechnology. Protease and phosphatase inhibitor cocktails were purchased from Roche (4693124001/4693159001). Dulbecco’s Modified Eagle’s Medium (DMEM), fetal bovine serum (FBS) and penicillin and streptomycin (PS) were purchased from Gibco. Polyvinylidene fluoride (PVDF) membranes were purchased from Millipore. The antibodies for immunoblot analysis are listed as follows: DJ-1 (Abcam, ab18257, 1:2500), LAMP2A (Abcam, ab18528, 1:1000), HSC70 (Abcam, ab1427/ab2788, 1:1000), SQSTM1/p62 (abcam, ab56416, 1:1000), α-synuclein (BD, 610787, 1:1000), α-synuclein (Invitrogen, AHB0261, 1:1000), LAMP-1 (CST, 3243S, 1:1000), GAPDH (CST, 2118S, 1:2500), LC3B (CST, 3868S, 1:1000), COX IV (CST, 4805, 1:1000), c-Raf (CST, 12552S, 1:1000), actin (Sigma, A5441, 1:2500) and horseradish peroxidase-conjugated secondary antibodies (Jackson ImmunoResearch Laboratories, 115-035-144/146, 1:10000). All other chemicals were from Sigma-Aldrich unless otherwise specified.

### Cell Culture

Human neuroblastoma SH-SY5Y cells were obtained from the American Type Culture Collection and cultured in DMEM plus 10% FBS and 100 U/mL PS at 37°C in a humidified incubator with 5% CO_2_. For pharmacological studies, NH_4_Cl, CQ, 3-MA, lactacystin and CHX were added at the indicated times and concentrations.

### Transfection of Plasmid DNA and siRNA

Human WT α-synuclein and WT DJ-1 cDNAs were cloned into a pcDNA3.1-flag vector (Gui et al., 2012) and pcDNA3-HA vector (Wang et al., 2011) respectively. Lipofectamine 2000 was used to transfect SH-SY5Y cells with plasmid DNA and/or siRNA in accordance with the manufacturer’s instructions. Briefly, the cells were cultured in a 6-well plate to 80%–90% confluence, and transfected with the indicated amounts of DNA and/or siRNA. Transfection efficiency was verified by Western blotting 48 h post-transfection.

### Western Blotting Analysis

Protein extraction and Western blotting were carried out as previously described (Jiang et al., 2015). The cells were lysed in RIPA buffer (50 mM Tris-HCl pH 8.0, 150 mM NaCl, 0.1% SDS, 0.5% sodium deoxycholate and 1% NP-40) supplemented with protease and phosphatase inhibitor cocktails and 1 mM PMSF for 30 min on ice. After centrifugation at 14,000 *g* for 15 min at 4°C, the protein concentration in the supernatants was determined with a BCA protein assay kit (Thermo Scientific, 23225). Equal amounts of protein (20 μg/lane) were separated by SDS-PAGE and then transferred to PVDF membranes. The membranes were incubated in blocking buffer for 1 h at room temperature and subsequently incubated with primary antibodies overnight at 4°C. The membranes were then incubated with horseradish peroxidase-conjugated secondary antibodies for 1 h at room temperature. Finally, the signals were detected by chemiluminescence (BIO-RAD ChemiDoc MP Imaging System) according to the manufacturer’s instructions.

### Quantitative Real-time PCR

Total RNA was extracted from cultured cells using TRIzol reagent (Invitrogen). Reverse transcription was carried out using PrimerScript Reverse Transcriptase. Real-time quantitative PCR was performed using Roche LightCycler^®^ 480 system with SYBR Green reagents (Takara). The results were determined by the 2^−ΔΔCt^ method to compare gene expression with *GAPDH* expression as a loading control. Primer sequences were as follows: *LAMP2A* forward: 5′-GCACAGTGAGCACAAATGAGT-3′; reverse: 5′-CAGTGGTGTGTATGGTGGGT-3′. GAPDH forward: 5′-GCACAGTGAGCACAAATGAGT-3′, reverse: 5′-CAGTGGTGTGTATGGTGGGT-3′.

### Isolation of Lysosomes

Lysosomes from human neuroblastoma SH-SY5Y cells were isolated with Lysosome Enrichment Kit for Tissue and Cultured Cells (Thermo Scientific, 89839) following manufacturer’s instructions. The kit uses OptiPrep Cell Separation Media for the density-based separation of lysosomes from contaminating cell structures. After three washes with PBS, the cultured cells were homogenized with extraction buffer from a Lysosome Enrichment Kit. Then, the lysosomes were isolated by density gradient centrifugation (145,000× *g* for 2 h). For the assessment of cyt-HSC70, 30 μl volume of the incubation buffer (10 mM MOPS, pH 7.3, 0.3 M sucrose) supplemented with protease and phosphatase inhibitor cocktails and 1 mM PMSF was added into the freshly isolated intact lysosomes, and then proteinase K (5 μl of a 1 mg/ml stock in 1 mM CaCl_2_, 50 mM Tris-HCl, pH 8) was added into the incubation buffer for 10 min at 0°C (Kaushik and Cuervo, 2009). After that, 1 ml volume of incubation buffer supplemented with protease and phosphatase inhibitor cocktails and 1 mM PMSF were added and centrifuged all samples at 25,000 *g* for 5 min at 4°C (Kaushik and Cuervo, 2009). Aspirate the supernatant fractions and wash the pellet fractions twice with incubation buffer to eliminate any protein bound non-specifically to the lysosome surface (Kaushik and Cuervo, 2009). Finally, the lysosomes were lysed in RIPA buffer, assayed by Western blotting for the levels of LAMP1, c-Raf and COX IV and used in the following experiments. A total of 5 μg of protein in lysosome-enriched fractions was loaded into each lane according to the protein concentration measured.

### Animals

DJ-1 knockout mice (kindly provided by Dr. Jie Shen, Harvard Medical School) were back-crossed with C57BL/6 mice at least 6 times. The mice were housed 5 per cage in a controlled environment under 22–25°C with 40%–60% relative humidity, and a 12 h light/dark cycle with free access to food and water. The study was approved by the Ethics Committee of Ruijin Hospital affiliated to Shanghai Jiao Tong University School of Medicine, and all procedures were carried out in accordance with the guidelines of the laboratory animal ethical standards of Shanghai Jiao Tong University School of Medicine.

### MPTP Administration

DJ-1 knockout and WT littermate male mice (8–10 weeks old) used in the experiments received MPTP as previously described (Jackson-Lewis and Przedborski, 2007). In brief, the mice were treated with MPTP (30 mg/kg/day, intraperitoneally) once a day for five consecutive days, while control mice were injected with saline only (Jackson-Lewis and Przedborski, 2007). At the indicated times (0, 4, 7, 14 and 21 days) after the last MPTP injection, the mice were sacrificed. The ventral midbrain tissue was isolated, weighed and then stored at −80°C until used as described (Jackson-Lewis and Przedborski, 2007).

### α-Synuclein Sequential Extraction

The procedures were carried out as previously described (Roberts et al., 2015) with minor modifications. For the sequential extraction, the following buffers were used: (1) TBS buffer: 50 mM Tris-HCl pH 7.4, 175 mM NaCl and 5 mM EDTA; (2) TBST buffer: TBS with 1% Triton X-100; (3) RIPA buffer (SDS buffer): 50 mM Tris-HCl pH 7.4, 175 mM NaCl, 5 mM EDTA, 1% NP-40, 0.5% sodium deoxycholate and 0.1% SDS; and (4) Urea buffer: 8 M urea/5% SDS. Ventral midbrain tissue was homogenized in sequence with the four sequential extraction buffers listed above and supplemented with cocktails and PMSF to generate TBS, TBST, SDS and urea-soluble fractions. First, the sample was homogenized in twenty volumes of TBS buffer on ice. The homogenate was centrifuged for 5 min at 1000 *g* at 4°C, and then the supernatant was ultracentrifuged for 30 min at 120,000 *g* at 4°C. The resulting supernatant was the TBS-soluble fraction. Second, the pellet was washed twice in TBS buffer, resuspended by sonication in TBST buffer and centrifuged for 20 min at 120,000 *g* at 4°C. The resulting supernatant was the TBST-soluble fraction. Third, the pellet was washed twice, resuspended by sonication in RIPA buffer and centrifuged for 20 min at 120,000 *g* at 4°C. The resulting supernatant was the SDS soluble fraction. Fourth, the pellet was washed twice with TBS buffer at room temperature and then resuspended by sonication in urea buffer for the urea-soluble fraction.

### Statistical Analysis

All values are expressed as the mean ± SEM. Data were from three or more independent experiments. The relative intensities were normalized to internal loading control and further normalized to the control levels. Statistical analysis was performed using Prism 5 (GraphPad Software). Statistical significance was determined by one-way analysis of variance (ANOVA) and two-way ANOVA with Bonferroni’s *post hoc* test. Values of *p* < 0.05 were considered statistically significant.

## Results

### DJ-1 Deficiency Accelerated α-Synuclein Accumulation and Aggregation in SH-SY5Y Cells and PD Animal Models

**Figure 1:**
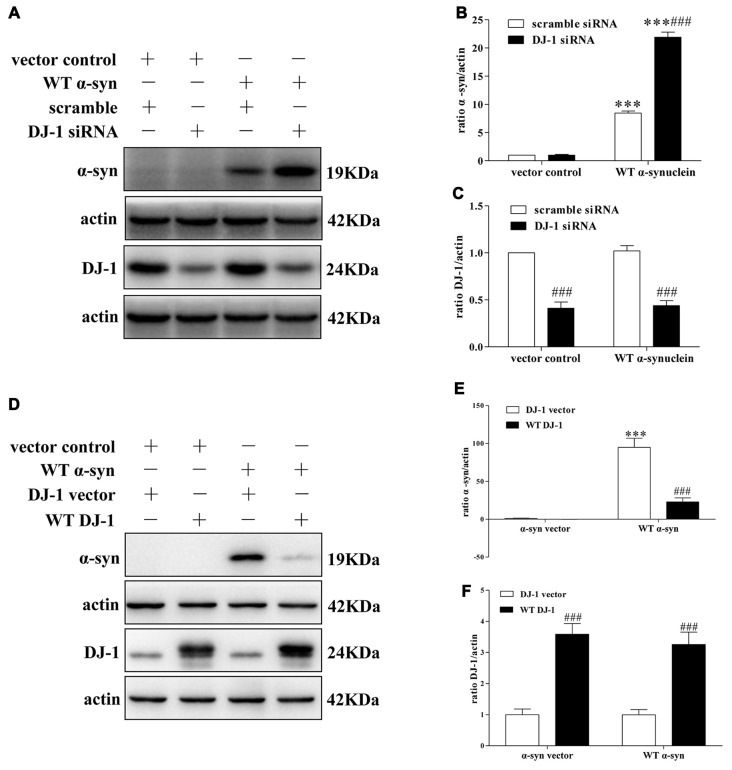
DJ-1 inhibits α-synuclein accumulation in SH-SY5Y cells. **(A–C)** DJ-1 deficiency aggravated the accumulation of α-synuclein in SH-SY5Y cells. Wild-type (WT) α-synuclein plasmid and DJ-1 siRNA were co-transfected into SH-SY5Y cells for 48 h. Immunoblots for the indicated proteins are shown in **(A)**, and quantifications of changes in the levels of α-synuclein and DJ-1 are shown in **(B,C)** respectively (mean ± SEM, *n* = 3, ***p < 0.001 vs. α-synuclein vector control, ^###^*p* < 0.001 vs. scrambled control). **(D–F)** WT DJ-1 overexpression inhibited accumulation of α-synuclein in SH-SY5Y cells. WT α-synuclein plasmid and WT DJ-1 plasmid were co-transfected into SH-SY5Y cells for 48 h. Immunoblots for the indicated proteins are shown in **(D)**, and quantifications of changes in the levels of α-synuclein and DJ-1 are shown in **(E,F)** respectively (mean ± SEM, *n* = 6, ***p < 0.001 vs. α-synuclein vector control, ^###^*p* < 0.001 vs. DJ-1 vector control).

To explore the underlying molecular mechanisms, we established a cellular system transiently co-transfected with siRNA against DJ-1 and WT α-synuclein plasmid into SH-SY5Y cells, and the transfection efficiency was verified by Western blotting (Supplementary Figure S1). In agreement with previous research (Shendelman et al., 2004), Western blotting analysis confirmed that DJ-1 deficiency markedly accelerated α-synuclein accumulation (Figures 1A–C), while DJ-1 overexpression inversely inhibited α-synuclein accumulation in SH-SY5Y cells (Figures 1D–F).

**Figure 2:**
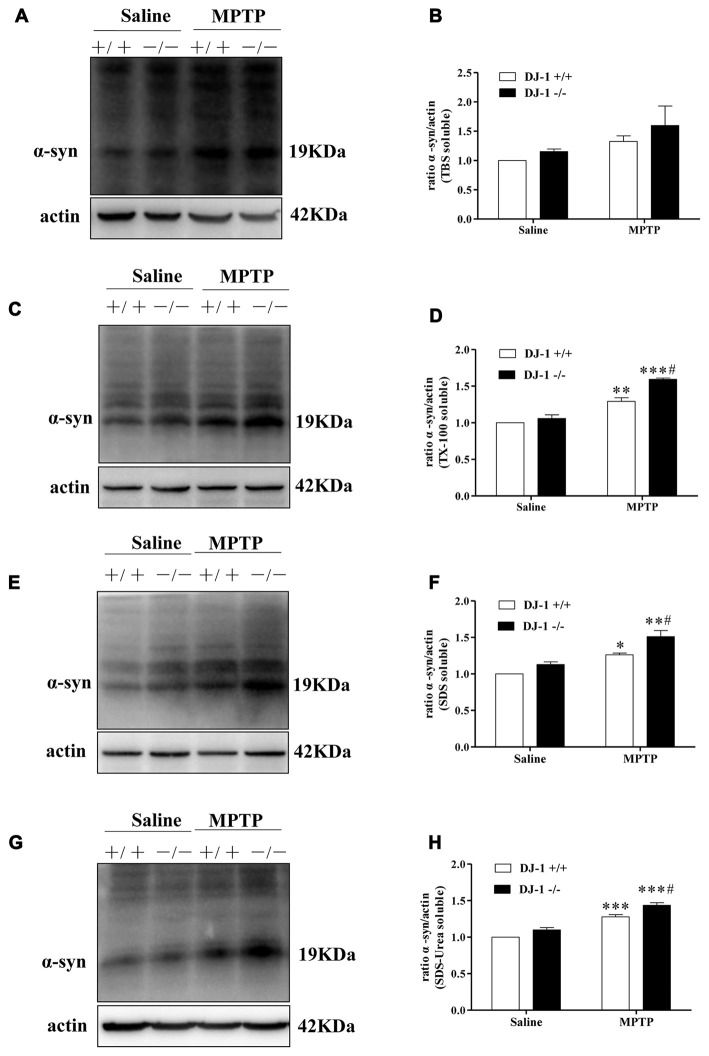
DJ-1 inhibits the accumulation and aggregation of α-synuclein in Parkinson’s disease (PD) animal models. **(A–H)** DJ-1 deficiency accelerates the accumulation and aggregation of α-synuclein in DJ-1−/− mouse ventral midbrain samples after Methyl-4-phenyl-1,2,3,6-tetrahydropyridine (MPTP) administration in the SDS-soluble and urea-soluble fraction compared with that of DJ-1+/+ mice. Immunoblots for α-synuclein are shown in **(A)** for TBS-soluble, **(C)** for TX-100-soluble, **(E)** for SDS-soluble and **(G)** for urea-soluble fractions. In addition, quantifications of changes in α-synuclein levels are shown in **(B,D**,**F,H)** (mean ± SEM, *n* = 3, *p < 0.05, **p < 0.01, ***p < 0.001 vs. saline control; ^#^*p* < 0.05 vs. DJ-1+/+).

Furthermore, we studied the relationship between DJ-1 and α-synuclein in PD animal models (Figures 2A–H). In this experiment, we established a MPTP mouse model of PD in DJ-1 knockout and WT littermate mice to determine the effect of DJ-1 deficiency on α-synuclein aggregation *in vivo*. First, WT mice received free base MPTP 30 mg/kg daily for five consecutive days and were sacrificed at different times to analyze the changes in α-synuclein levels in ventral midbrain regions. Consistent with previous research (Vila et al., 2000), α-synuclein total tissue protein peaked at day 4 after MPTP administration and then returned to control level until 21 days (Supplementary Figure S2). Second, to further determine whether the effect of DJ-1 deficiency on α-synuclein aggregation with or without MPTP administration, we analyzed α-synuclein levels in DJ-1 knockout and WT mice at day 4 after administration (Figures 2A–H). In agreement with the previous results (Yamaguchi and Shen, 2007), there was no significant difference between the two groups that received salinein all the fractions (Figures 2A–H), whereas soluble and insoluble α-synuclein oligomers in DJ-1 knockout mice that received MPTP increased more prominently in the SDS and urea-soluble fractions compared with those in WT control mice (Figures 2E–H). Taken together, our results showed that DJ-1 deficiency aggravated accumulation and aggregation of α-synuclein in both SH-SY5Y cells and PD animal models.

### α-Synuclein Degradation via the CMA Pathway in SH-SY5Y Cells

**Figure 3:**
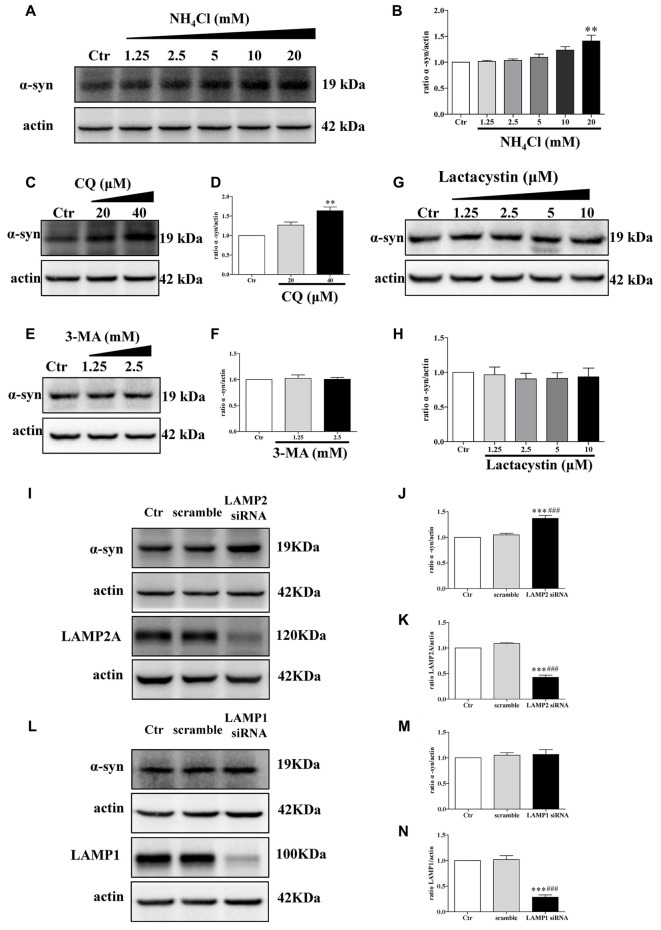
Endogenous α-synuclein is degraded in lysosomes by chaperone-mediated autophagy (CMA) in SH-SY5Y cells. **(A,B)** Levels of endogenous α-synuclein were determined in SH-SY5Y cells treated with NH_4_Cl for 9.5 h. Immunoblots for the indicated proteins are shown in **(A)** and quantification of α-synuclein levels is shown in (**B**; mean ± SEM, *n* = 3, **p < 0.01 vs. control). **(C–H)** Levels of endogenous α-synuclein were determined in SH-SY5Y cells treated with Chloroquine diphosphate (CQ), 3-methyladenine (3-MA) and lactacystin respectively for 24 h. Immunoblots for the indicated proteins are shown in **(C**,**E,G)**, and quantifications of α-synuclein levels are shown in **(D**,**F**,**H)** respectively (mean ± SEM, *n* = 3, **p < 0.01 vs. control). **(I–K)** Levels of endogenous α-synuclein were determined in lysosome-associated membrane protein type-2 (LAMP2) knockdown SH-SY5Y cells. Immunoblots for the indicated proteins are shown in **(I)**, and the quantifications of α-synuclein and LAMP2A levels are shown in **(J,K)** respectively (mean ± SEM, *n* = 3, ***p < 0.001 vs. control, ^###^*p* < 0.001 vs. scramble). **(L–N)** Levels of endogenous α-synuclein were determined in LAMP1 knockdown SH-SY5Y cells. Immunoblots for the indicated proteins are shown in **(L)**, and the quantifications of α-synuclein and LAMP1 levels are shown in **(M,N)** respectively (mean ± SEM, *n* = 3, ***p < 0.001 vs. control, ^###^*p* < 0.001 vs. scramble).

The proteolytic pathways of a given protein are determined by cell type, cellular conditions, or activity of the pathway (Park and Cuervo, 2013). To determine whether WT α-synuclein can be degraded by the lysosomal pathway in our cellular system, we treated SH-SY5Y cells with NH_4_Cl or CQ (inhibitors of lysosomes) at different concentrations. Endogenous α-synuclein levels increased after administration with NH_4_Cl or CQ (Figures 3A–D), whereas administration with lactacystin (a proteasome inhibitor) in SH-SY5Y cells failed to upregulate α-synuclein levels (Figures 3G,H), suggesting the lysosomal pathway is the primary proteolytic pathway for endogenous α-synuclein but not proteasome. In accordance with a previous study (Cuervo et al., 2004), α-synuclein was mainly degraded in lysosomes by CMA but not macroautophagy. Because endogenous α-synuclein levels were augmented when LAMP2 was knocked down in SH-SY5Y cells but not LAMP1, a known marker for lysosome (Figures 3I–N), whereas 3-MA at low concentrations (an inhibitor of macroautophagy) had little effect on the levels of α-synuclein (Figures 3E,F). The transfection efficiency of siRNA against LAMP2 and LAMP1, and the role of 3-MA in macroautophagy were determined by Western blotting (Supplementary Figure S3). These results suggested that CMA is the primary degradation pathway for endogenous WT α-synuclein in our cellular system and the accumulation of α-synuclein in DJ-1-deficient cells may result from the inhibition of CMA-dependent degradation of α-synuclein.

### DJ-1 Inhibited α-Synuclein Accumulation by the CMA Pathway

**Figure 4:**
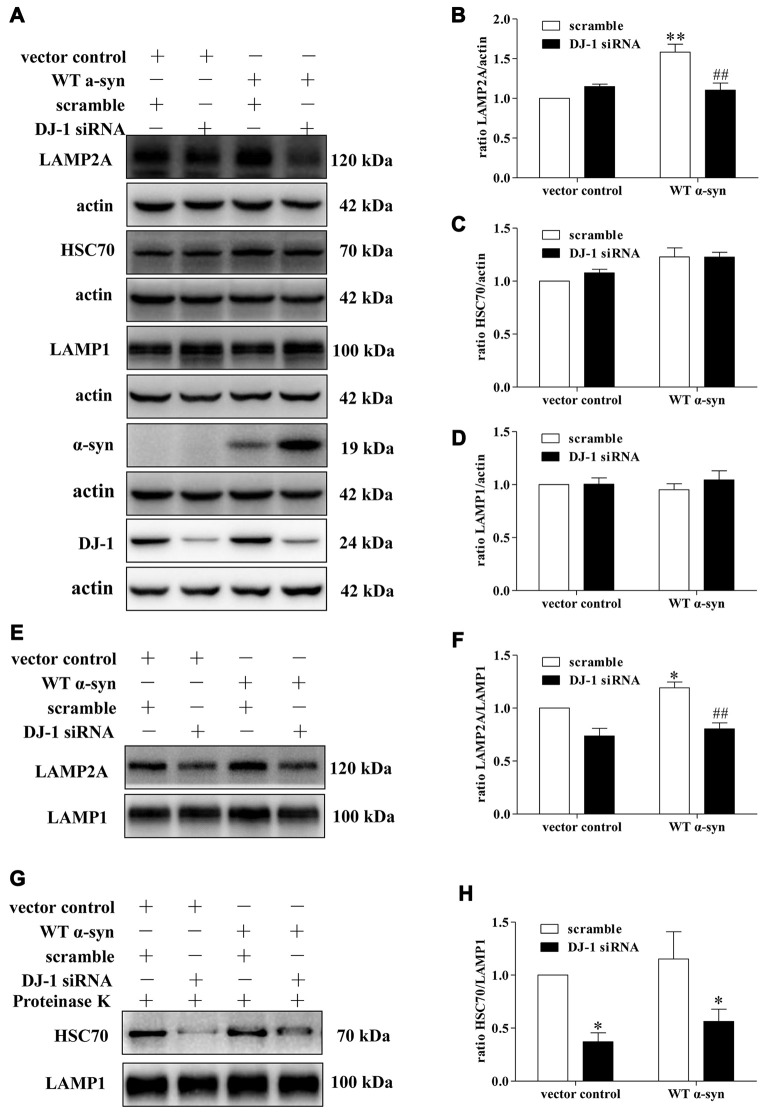
Effects of DJ-1 deficiency on CMA and α-synuclein levels in SH-SY5Y cells. **(A–D)** Levels of LAMP2A, HSC70, LAMP1, α-synuclein and DJ-1 were determined in homogenates of SH-SY5Y cells co-transfected with WT α-synuclein plasmid and DJ-1 siRNA. Immunoblots for the indicated proteins are shown in **(A)**, and quantification of the levels of LAMP2A, HSC70 and LAMP-1 are shown in **(B–D)** respectively (mean ± SEM, *n* = 3, 3, 4, **p < 0.01 vs. vector control, ^##^*p* < 0.01 vs. scramble). **(E–H)** Levels of LAMP2A, HSC70 and LAMP-1 in lysosome-enriched fractions isolated from SH-SY5Y cells co-transfected with WT α-synuclein plasmid and DJ-1 siRNA were determined. Immunoblots for the indicated proteins are shown in **(E,G)**, and quantifications of the levels of LAMP2A and HSC70 are shown in **(F,H)** respectively (mean ± SEM, *n* = 4, *p < 0.001 vs. vector control, ^##^*p* < 0.01 vs. scramble).

Since LAMP2A acts as both the receptor and essential components of the CMA translocation complex, LAMP2A levels at the lysosomal membrane are directly proportional to CMA activity (Schneider and Cuervo, 2013; Patel and Cuervo, 2015). Mild WT α-synuclein overexpression upregulated the total cellular levels of LAMP2A, but this upregulation was inhibited in DJ-1-deficient cells (Figures 4A,B). Meanwhile, the downregulation of LAMP2A was accompanied by an upregulation of α-synuclein in DJ-1-deficient cells (Figure 4A). Therefore, DJ-1 deficiency may accelerate α-synuclein accumulation by inhibiting the upregulation of LAMP2A under the condition of mild WT α-synuclein overexpression. To further confirm the role of DJ-1 deficiency in LAMP2A levels at the lysosomal membrane, we examined the levels of LAMP2A in the lysosomal fractions isolated from SH-SY5Y cells in the same cellular system (Figures 4E,F). The purity of lysosomal fractions was verified by Western blotting for LAMP1 (the lysosome marker), c-Raf (the cytoplasmic marker) and COX IV (the mitochondria marker) in both homogenate and lysosome-enriched fractions (Supplementary Figure S4). Consistent with the total whole cell lysate results, DJ-1 deficiency suppressed the increase in LAMP2A levels induced by mild WT α-synuclein overexpression in the lysosomal fraction (Figures 4E,F). In addition, there were no significant differences in LAMP1 levels among the groups (Figures 4A,D), suggesting a selective effect of DJ-1 on LAMP2A.

We then analyzed the effect of DJ-1-deficiency on HSC70 levels of in our established cellular system, because only HSC70-positive lysosomes are capable of performing CMA (Cuervo et al., 1997). WT α-synuclein overexpression slightly upregulated the levels of cyt-HSC70, but there was no obvious difference (Figures 4A,C), while DJ-1 deficiency strongly decreased the levels of lys-HSC70 compared with those in the control group (Figures 4G,H). Since HSC70 is a constitutively expressed chaperone protein and lys-HSC70 accounts for only a small proportion, the increase in cyt-HSC70 is not informative regarding CMA activity (Agarraberes et al., 1997; Patel and Cuervo, 2015). Therefore, DJ-1 may play an important role in the activation of CMA induced by mild WT α-synuclein accumulation and DJ-1 deficiency may decrease CMA-mediated degradation of α-synuclein by downregulating the key CMA components.

### The Mechanism of DJ-1 Affected the Levels of LAMP2A in Lysosomes

**Figure 5:**
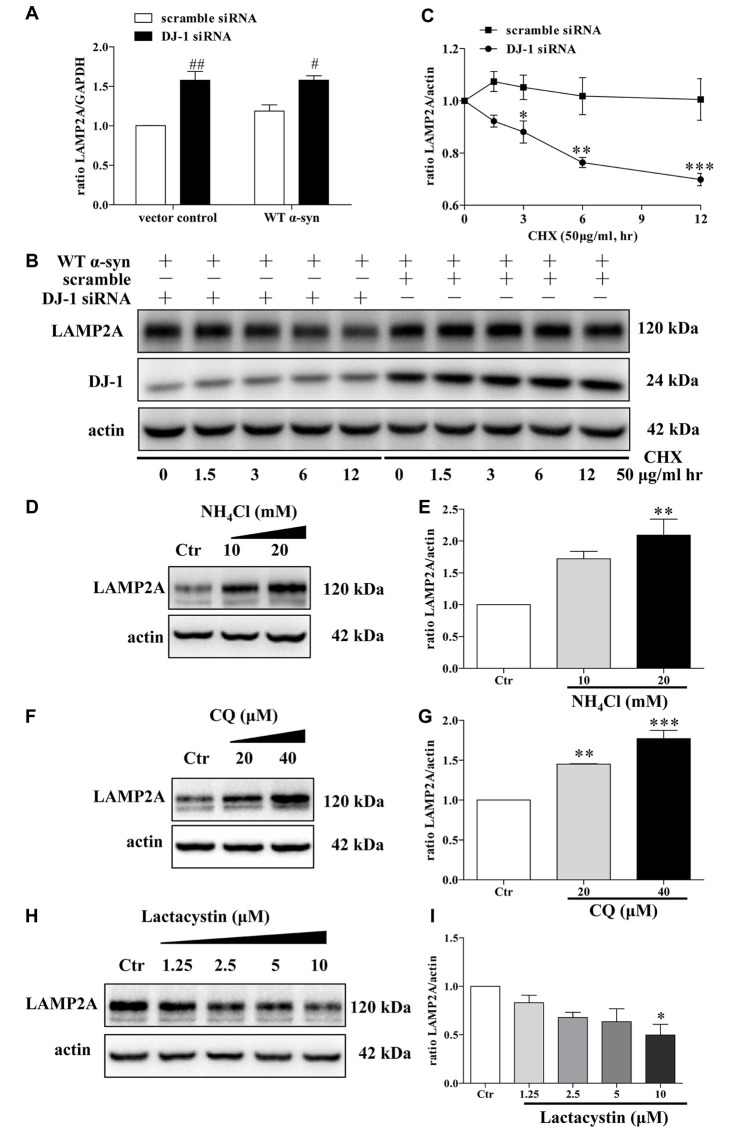
Accelerated LAMP2A degradation in lysosomes in DJ-1-deficient SH-SY5Y cells. **(A)** The levels of LAMP2A mRNAs in SH-SY5Y cells co-transfected with WT α-synuclein plasmid and DJ-1 siRNA for 48 h were analyzed by real-time PCR analysis (mean ± SEM, *n* = 3, ^#^*p* < 0.05 vs. scramble, ^##^*p* < 0.01 vs. scramble). **(B,C)** The degradation of LAMP2A is accelerated in DJ-1-deficient cells. SH-SY5Y cells were co-transfected with WT α-synuclein plasmid and DJ-1 siRNA or scramble for 48 h and then incubated with cycloheximide (CHX; 50 μg/ml) for the indicated times. Immunoblots for the indicated proteins are shown in **(B)** and quantification of LAMP2A levels is shown in (**C**; mean ± SEM, *n* = 4, *p < 0.05 vs. scramble, **p < 0.01 vs. scramble, ***p < 0.001 vs. scramble). **(D–G)** Levels of endogenous LAMP2A were determined in SH-SY5Y cells treated with NH_4_Cl, CQ and lactacystin for 24 h. Immunoblots for the indicated proteins are shown in **(D,F,H)**, and quantifications of LAMP2A levels are shown in **(E,G,I)** respectively (mean ± SEM, *n* = 3, *p < 0.05 vs. control, **p < 0.01 vs. control, ***p < 0.001 vs. control).

Since LAMP2A is the rate-limiting step in the CMA pathway and it is tightly regulated by *de novo* synthesis and degradation at the lysosomal membrane (Kaushik and Cuervo, 2012), we thus investigated LAMP2A reduction in DJ-1-deficient cells in the above mentioned contexts. DJ-1 deficiency upregulated LAMP2A mRNA levels, suggesting that LAMP2A reduction induced by DJ-1 deficiency was not due to *de novo* synthesis inhibition (Figure 5A). We then evaluated whether LAMP2A downregulation was due to enhanced degradation of LAMP2A by the protein synthesis inhibitor CHX to exclude the effect of translation. Compared with the control, DJ-1 deficiency notably increased the rate of degradation for LAMP2A (Figures 5B,C). In line with previous research (Cuervo and Dice, 2000a), our results showed that LAMP2A was degraded in lysosomes but not the ubiquitin-proteasome system (Figures 5D–I). In brief, DJ-1 deficiency may aggravate the accumulation of α-synuclein by accelerating the degradation of LAMP2A in lysosomes.

## Discussion

**Figure 6:**
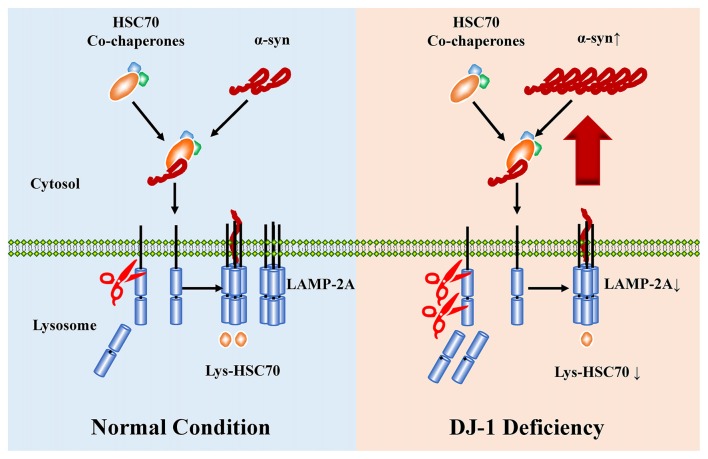
The proposed working model for DJ-1 in suppressing α-synuclein accumulation and aggregation by regulating CMA. WT a-synulcien is a substrate of CMA in normal conditions, whereas DJ-1 deficiency accelerated the degradation of LAMP2A and impaired the activity of CMA, leading to α-synuclein accumulation.

Loss-of-function mutations in DJ-1 gene cause autosomal recessive early-onset familial PD (Bonifati et al., 2003), a neurodegenerative movement disorder pathologically characterized by the presence of α-synuclein-containing Lewy bodies in surviving dopaminergic neurons (Spillantini et al., 1997, 1998; Lees et al., 2009). DJ-1 can inhibit α-synuclein aggregation (Shendelman et al., 2004), but the detailed mechanisms remain unclear. Here, DJ-1 deficiency accelerated the degradation of LAMP2A in lysosomes, which is the rate-limiting step in the CMA pathway, leading to α-synuclein accumulation (Figure 6).

DJ-1 can inhibit α-synuclein aggregation (Shendelman et al., 2004; Zondler et al., 2014), and immunohistology has revealed that DJ-1 is present in the halo part of Lewy bodies, which contain insoluble α-synuclein, in sporadic PD patients (Neumann et al., 2004; Jin et al., 2005). Our results showed that DJ-1 deficiency aggravated accumulation and aggregation of α-synuclein in both SH-SY5Y cells and PD animal models. The tendency of the changes in α-synuclein levels in PD animal models is in line with previous behavioral studies (Kim et al., 2005; Takahashi-Niki et al., 2015). However, whether DJ-1 directly interacts with α-synuclein is a controversial topic. Zondler et al. (2014) have reported that DJ-1 could interact directly with α-synuclein, thus decreasing α-synuclein aggregation and toxicity in PD models, whereas the direct interaction between DJ-1 and α-synuclein was not observed in another investigation (Jin et al., 2007). Shendelman et al. (2004) reported that DJ-1 does not colocalize with α-synuclein protein aggregates, suggesting that DJ-1 may play an inhibitory role during the early stages of α-synuclein aggregates consisting of misfolded monomers but not the oligomeric species, or even inclusion bodies, while WT soluble α-synuclein is mainly degraded by CMA (Cuervo et al., 2004; Vogiatzi et al., 2008). In our study, DJ-1 deficiency aggravated α-synuclein aggregation by regulating the CMA pathway.

CMA is responsible for energy balance and protein homeostasis under normal physiological conditions (Cuervo and Wong, 2014), whereas CMA dysfunction is closely associated with both sporadic (Martinez-Vicente et al., 2008; Alvarez-Erviti et al., 2010; Orenstein et al., 2013) and familial (Cuervo et al., 2004; Kabuta et al., 2008; Orenstein et al., 2013; Tang et al., 2015) PD. Taken together with our research, all of these observations support the idea that several familial PD gene mutations, such as A53T and A30P α-synuclein mutants (Cuervo et al., 2004), I93M UCH-L1 mutant (Kabuta et al., 2008), G2019S LRRK2 mutant (Orenstein et al., 2013) and VPS35 deficiency or mutation (Tang et al., 2015), impair the normal function of the LAMP2A translocation complex and converge at the CMA pathway, blocking CMA substrates degradation and aggravating α-synuclein accumulation and aggregation, which is thought to play a central role in PD pathogenesis (Cuervo and Wong, 2014).

DJ-1 has multiple functions regulated by the oxidative state (Ariga et al., 2013). DJ-1 includes three cysteine residues (C46, C56, and C106), and C106 is especially vulnerable to oxidative stress and is sequentially oxidized to SOH, SO_2_H and SO_3_H (Ariga et al., 2013). DJ-1 at C106 with SO_2_H is considered an active form, whereas excessive oxidation of DJ-1 (with SO_3_H) renders DJ-1 inactive in patients with sporadic PD, thus providing evidence that DJ-1 participates in the onset and pathogenesis of sporadic PD and familial PD (Ariga et al., 2013). Therefore, we speculated that similar to the case of PD-related DJ-1 mutations, some post-translationally modified forms of DJ-1, such as DJ-1 SO_3_H, may also participate in the pathogenesis of sporadic PD by decreasing the levels of LAMP2A and subsequently decreasing the levels of CMA. This hypothesis may explain the α-synuclein aggregation in WT mice treated with MPTP in our research. However, whether the changes in these post-translational modifications can reduce LAMP2A levels and CMA activity requires further investigation.

There is a close relationship between DJ-1 and CMA. DJ-1 is a CMA substrate, and CMA can regulate mitochondrial function via regulating DJ-1 homeostasis (Wang et al., 2016). Since cytosolic substrates compete for lysosomal uptake and degradation by CMA (Kaushik and Cuervo, 2009), the absence of DJ-1 could promote the degradation of other CMA substrates. However, DJ-1 decreased the levels of α-synuclein (a well-characterized CMA substrates) by regulating CMA in our study. This finding suggests a complex relationship between DJ-1 and CMA, and the result may depend on the overall effect of multiple molecular pathways in different cellular contexts.

CMA activity was positively correlated with lysosomal LAMP2A levels, which are the primary target for CMA regulation (Cuervo and Dice, 2000a). In most instances, LAMP2A levels are directly regulated by degradation at the lysosomal membrane (Cuervo and Dice, 2000a,b) but not by *de novo* synthesis (Kiffin et al., 2004). LAMP2A reduction was induced by the acceleration of the degradation rate in lysosomes in DJ-1-deficient cells. LAMP2A monomers are degraded in lysosome membrane lipid microdomains (Cuervo et al., 2003), and many factors may be involved in the regulation, such as GFAP, EF1α and the fluidity of the lysosomal membrane (Cuervo and Wong, 2014). Alterations in the lipid composition at the lysosomal membrane may play an important role in reduced CMA activity by influencing the stability of lysosomal LAMP2A (Rodriguez-Navarro and Cuervo, 2012; Rodriguez-Navarro et al., 2012). However, information regarding the effect of DJ-1 on lipid metabolism is still relatively limited. DJ-1 deficiency enhances the levels of serum low-density lipoprotein cholesterol by transcriptional regulation of the low-density lipoprotein receptor gene (Yamaguchi et al., 2012). Further studies are needed to determine whether DJ-1 can regulate the degradation of LAMP2A at the lysosomal membrane by regulating lipid metabolism.

In summary, our research showed that DJ-1 deficiency increased the accumulation of α-synuclein by accelerating the degradation of LAMP2A in lysosomes. Our study provides further evidence for the interplay between PD-associated proteins and has an important implication for understanding of the biology of DJ-1 in the pathogenesis of PD.

## Author Contributions

S-DC, JL and J-QD: conception and design of the study; C-YX, W-YK, Y-MC, T-FJ, JZ and L-NZ: data acquisition and analysis; C-YX, S-DC and JL: and drafting a significant portion of the manuscript or figures.

## Conflict of Interest Statement

The authors declare that the research was conducted in the absence of any commercial or financial relationships that could be construed as a potential conflict of interest.
