# Supplementary material for: DJ-1 Inhibits α-Synuclein Aggregation by Regulating Chaperone-Mediated Autophagy
Source: Front Aging Neurosci. 2017 Sep 27;9:308. doi: 10.3389/fnagi.2017.00308 (PMC5623690; doi:10.3389/fnagi.2017.00308)
Supplement: Supplementary file 1 [file Data_Sheet_1.docx]

**
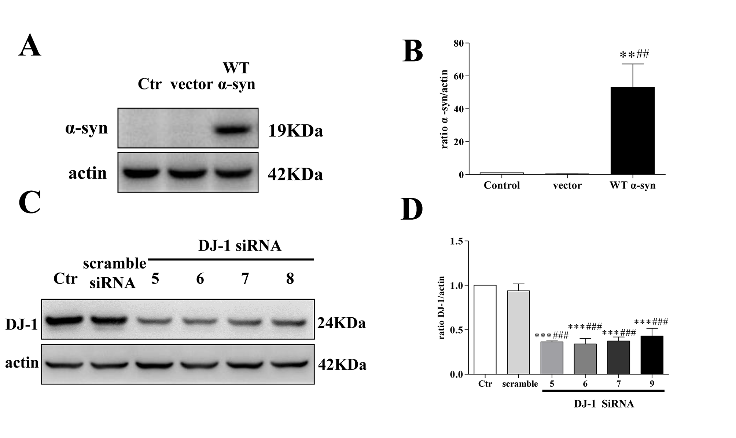
**

**Supplementary Figure 1. The transfection efficiency of WT α-synuclein plasmid DNA and DJ-1 siRNA.**

(A-B) Exogenous WT α-synuclein was overexpressed in SH-SY5Y cells. Immunoblots for the indicated proteins are shown in (A) and quantification of α-synuclein levels is shown in (B) (mean ± SEM, n=4, ***p* < 0.01 vs control, ^##^*p* < 0.01 vs vector control). (C-D) DJ-1 was efficiently silenced with siRNA for 48 h in SH-SY5Y cells. Immunoblots for the indicated proteins are shown in (C), and quantification of DJ-1 levels is shown in (D) (mean ± SEM, n=3, ****p* < 0.001 vs control, ^###^*p* < 0.001 vs scrambled control).


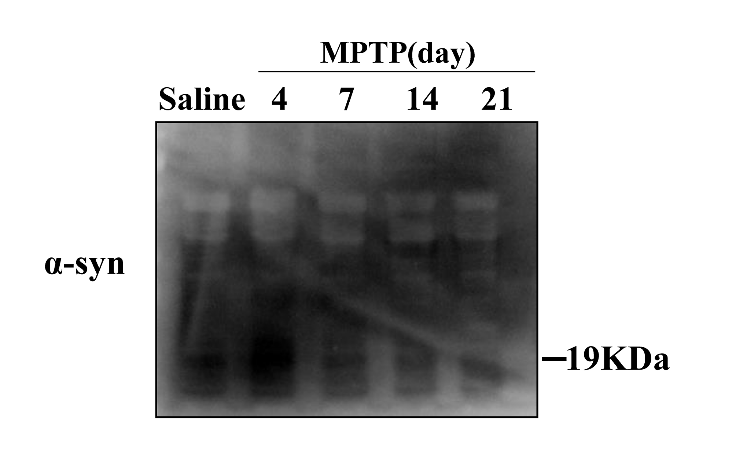


**Supplementary Figure 2. The effect of MPTP administration on α-synuclein levels in WT mice**

The amounts of α-synuclein in total tissue protein increased progressively in ventral midbrain samples of DJ-1+/+ mice from 0 to 4 days after MPTP administration and then returned to the level of controls. A total of 15 µg of total protein was loaded into each lane according to the protein concentration measured.

**
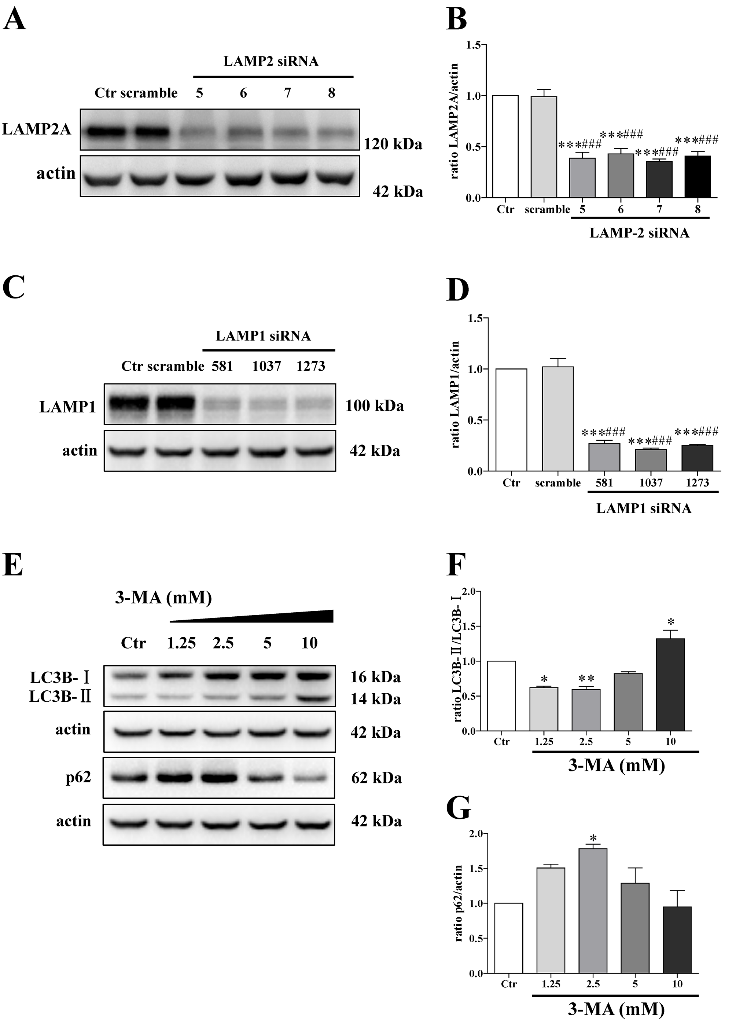
**

**Supplementary Figure 3. The transfection efficiency of siRNA against LAMP2 and LAMP1, and** **the dual role of 3-MA in macroautophagy**

(A-D) LAMP2A and LAMP1 were efficiently silenced with LAMP2 and LAMP1 siRNA respectively for 48 h in SH-SY5Y cells. Immunoblots for the indicated proteins are shown in (A,C), and the quantifications of LAMP2A and LAMP1 levels are shown in (B,D) respectively (mean ± SEM, n=3, ****p* < 0.001 vs control, ^###^*p* < 0.001 vs scrambled control. (E-G) Levels of LC3B and p62 were determined in SH-SY5Y cells treated with different concentrations of 3-MA for 24 h. 3-MA at low concentrations acted as an inhibitor of macroautophagy, whereas high concentrations of 3-MA inversely promoted macroautophagy. Immunoblots for the indicated proteins are shown in (E), and the quantifications of LC3B and p62 levels are shown in (F,G) respectively (mean ± SEM, n=3, **p* < 0.05 vs control, ***p* < 0.01 vs control).


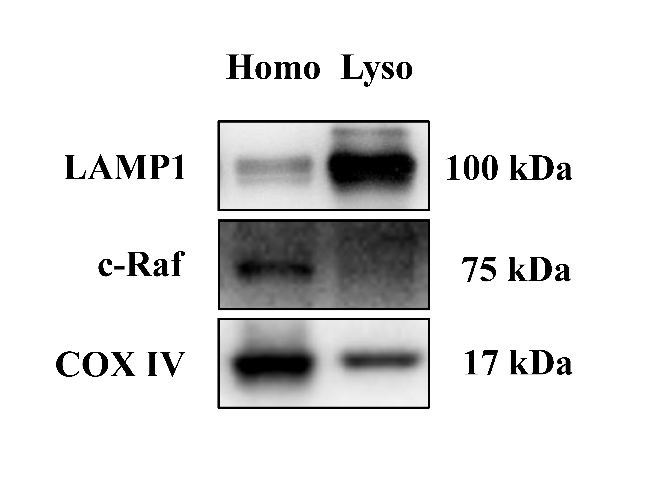


**Supplementary Figure 4. Assessment of purity of lysosomal fractions.**

The purity of lysosomal fractions was determined by Western blotting for the lysosome marker LAMP1, the cytoplasmic marker c-Raf and the mitochondria marker COX IV. A total of 5 µg of protein in homogenate (Homo) and lysosome-enriched fractions (Lyso) was loaded into each lane according to the protein concentration measured.
